# 4‐octyl Itaconate inhibits lipopolysaccharide (LPS)‐induced osteoarthritis via activating Nrf2 signalling pathway

**DOI:** 10.1111/jcmm.17185

**Published:** 2022-01-23

**Authors:** Qingchen Zhang, Xiaohui Bai, Rongrong Wang, Hao Zhao, Lili Wang, Jingwen Liu, Ming Li, Zheng Chen, Zejun Wang, Lianxin Li, Dawei Wang

**Affiliations:** ^1^ Department of Orthopaedics Shandong Provincial Hospital Cheeloo College of Medicine Shandong University Jinan China; ^2^ Department of Orthopaedics Shandong Provincial Hospital Affiliated to Shandong First Medical University Jinan China; ^3^ Department of Clinical Laboratory Shandong Provincial Hospital, Cheeloo College of Medicine Shandong University Jinan China

**Keywords:** 4‐octyl itaconate, lipopolysaccharide, Nrf2 signalling pathway, osteoarthritis

## Abstract

Small molecule drug intervention for chondrocytes is a valuable method for the treatment of osteoarthritis (OA). The 4‐octyl itaconate (OI) is a cellular derivative of itaconate with sound cell permeability and transformation rate. We attempted to confirm the protective role of OI in chondrocytes and its regulatory mechanism. We used lipopolysaccharide (LPS) to induce chondrocyte inflammation injury. After the OI treatment, the secretion and mRNA expression of *Il*‐*6*, *Il*‐*10*, *Mcp*‐*1* and *Tnf*‐*α* were detected by ELISA and qPCR. The protective effect of OI on articular cartilage was further verified in surgical destabilization of the medial meniscus model of OA. Cell death and apoptosis were evaluated based on CCK8, LDH, Typan blue staining, Annexin V and TUNEL analyses. The small interfering RNAs were used to knockout the *Nrf2* gene of chondrocytes to verify the OI‐mediated Nrf2 signalling pathway. The results revealed that OI protects cells from LPS‐induced inflammatory injury and attenuates cell death and apoptosis induced by LPS. Similar protective effects were also observed on articular cartilage in mice. The OI activated Nrf2 signalling pathway and promoted the stable expression and translocation of Nrf2 into the nucleus. When the Nrf2 signalling pathway was blocked, the protective effect of OI was significantly counteracted in chondrocytes and a mouse arthritis model. Both itaconate and its derivative (i.e., OI) showed important medical effects in the treatment of OA.

## 1 INTRODUCTION

Osteoarthritis (OA) is the most common joint disease worldwide and a common chronic disease in clinics. The pain and dysfunction caused by OA lead to not only physical damage to patients, but also severe economic burden to society.^1^, ^2^ The pathological changes of OA include synovial hyperplasia, articular cartilage destruction, osteophyte formation and subchondral ossification.^3^Articular cartilage is a type of hyaline cartilage, structurally composed of chondrocytes and matrix. Due to the lack of blood vessels, nerves and lymphoid tissues, the articular cartilage has limited ability to repair itself after acute or chronic injuries or joint degeneration. To date, there is no effective drug to improve the condition of OA, while the treatment is mainly to relieve the symptoms of patients with the surgical treatment as the only effective method to treat patients of OA at their late stages.^4^, ^5^, ^6^

For both the post‐traumatic arthritis and primary OA, the injury of chondrocytes caused by inflammatory reaction is closely related to the pathogenesis and development of OA.^7^, ^8^, ^9^ Lipopolysaccharide (LPS) is one of the structural components in the outer wall of Gram‐negative cells to provide a positive immune activation.^10^ Local injection of LPS into the joint induces not only synovitis, but also inflammatory injury of articular cartilage.^11^, ^12^, ^13^ The model of chondrocyte inflammation induced by LPS can effectively reflect the degree of the cartilage injury in OA.

In recent years, the understanding of metabolic physiology is advancing more rapidly than that of material and energy metabolisms. The disorder of one kind of metabolite can affect many signalling pathways.^14^, ^15^, ^16^, ^17^ Metabolic inflammation also plays a crucial role in the pathogenesis of OA, opening a new direction to explore potential treatments of OA.^18^ Furthermore, studies have shown that as an intermediate metabolite of tricarboxylic acid cycle (TCA cycle), itaconate plays important anti‐inflammatory and anti‐apoptotic roles. Specifically, itaconate weakens the succinate dehydrogenase (SDH) activity in cells, but not as effectively as traditional SDH inhibitors (e.g., sodium malonate), suggesting that itaconate may play an anti‐inflammatory role through different mechanisms.^19^ Itaconate and its derivatives have also been shown to regulate the IkBz‐ATF3 inflammatory axis.^20^ It has been confirmed that itaconate is a novel and effective activator of nuclear factor erythroid‐2‐related factor 2 (Nrf2), stabilizing and activating Nrf2 by alkylating cysteine residues on Keap1 and promoting the dissociation of Keap1‐Nrf2 complex. Itaconate is an α, β unsaturated carboxylic acid with electrophilicity. Hence, it can interact with proteins containing a thiol group at the cellular level, in a process called the electrophilic stress response (ESR).^21^ 4‐octyl itaconate has a ester group that allows it to infiltrate cells without the transporter protein. In addition, the electrophilic α, β unsaturated carboxylic acid of OI helps to bind to the Keap1 cysteine.^19^ Based on these characteristics, a novel type of itaconate derivative 4‐octyl itaconate (OI) was designed to effectively activate Nrf2 with anti‐inflammatory and protective roles in macrophages and other cells.^19^, ^22^, ^23^

Nuclear factor erythroid‐2‐related factor 2 is a crucial transcription factor mediating anti‐inflammation and anti‐apoptosis.^24^, ^25^, ^26^ Under physiological conditions, Nrf2 and Keap1 join to form polymers, regulated by Keap1.^19^, ^27^, ^28^ Once stimulated, the cysteine residue of Keap1 is modified, resulting in the release and accumulation of Nrf2 in the nucleus, binding with anti‐oxidative response element (ARE), and ultimately initiating the transcription and expression of a variety of anti‐inflammatory and anti‐apoptotic genes, including Glutamate‐Cysteine Ligase, Catalytic Subunit (*Gclc*), NADH Quinone Oxidoreductase 1 (*Nqo1*) and Haem Oxygenase‐1 (*Ho*‐*1*).^29^, ^30^ Furthermore, Nrf2 signalling pathway shows a strong anti‐inflammatory effect by limiting the progress of NF‐κB.^29^, ^31^

Studies have demonstrated that activation of Nrf2 inhibits cartilage destruction in models of OA.^32^, ^33^ Meanwhile, the Nrf2 knockout mice showed more severe cartilage damage than the wild‐type mice.^34^, ^35^ These results indicate that Nrf2 signalling pathway is involved in the formation and treatment of OA. Therefore, we hypothesize that activation of Nrf2 by itaconate under LPS‐induced inflammatory conditions in vitro may have a protective effect on chondrocyte‐associated inflammation, necrosis, and apoptosis in OA.

Itaconate activates Nrf2 signalling pathways with an indispensable effect on different cells or animal models, including the peripheral blood mononuclear cells of patients with systemic lupus erythematosus (SLE), human umbilical vein endothelial cells, neuronal cells, abdominal aortic aneurysms, osteoclasts and hepatic ischaemia‐reperfusion injury.^36^, ^37^, ^38^, ^39^, ^40^, ^41^ The role of itaconate in inflammatory injury of chondrocytes has not been studied, and the involvement of the Nrf2 signalling pathway in regulation of cartilage protection is still unclear. The purpose of this study is to investigate the effect and mechanism of OI on LPS‐induced inflammatory injury of articular cartilage. Our results confirm that OI inhibits LPS‐induced articular cartilage inflammatory injury by activating the Nrf2 signalling pathway.

## 2 METHODS

### 2.1 Reagents, chemicals and antibodies

Lipopolysaccharide was purchased from Sigma‐aldrich. OI and ML385 were synthesized by Topscience based on protocol described previously.^19^ NF‐κB p65 TF Assay Kit was purchased from Abcam. All mRNA primer sequences were synthesized by Biosune‐bio. Cell Counting Kit‐8 was purchased from Dojindo. Trypan Blue Staining and LDH cytotoxicity Assay Kits were acquired from Beyotime. PE Annexin V Apoptosis Detection Kit was purchased from BD‐bio. TUNEL dye and Lipofectamine 3000 were purchased from Thermo Fisher Scientific. Antibodies were acquired from Abcam and CST.

### 2.2 Cell culture and treatments

The mouse in vitro chondrocytes ATDC5 cell line was acquired from the RIKEN cell Bank (Tsukuba, Japan). Human normal chondrocytes C28I2 cell line were obtained from Otwo Biotech. Both cell lines were cultivated in DMEM containing 10% FBS at 37˚C with 5% CO_2_. After thawing, the cells were subcultured for more than three generations for further experiments. LPS was dissolved in dH_2_O to adjust its final concentration to 2 mg/ml. OI was dissolved in DMSO to make the final concentration as 25 mM. In the treatment of OI (0, 25, 50, 100 and 200 μM) for 12 h, the cells were cultured for 12 h after LPS (10 μg/ml) induction.

### 2.3 Enzyme‐linked immunosorbent assay

The mouse ATDC5 cells and human C28I2 cells were inoculated in 24‐well culture plates. After treated with LPS and OI, the supernatant was centrifuged for 10 min at 1000 *g* and 4°C. The samples were diluted twice and analysed according to the specifications of ELISA Kits, including Mouse Il‐6 ELISA Kit (VAL604, Novus), Mouse Mcp‐1 ELISA Kit (ab100721, Abcam), Mouse Il‐10 ELISA Kit (VAL605, Novus), Mouse Tnf‐α ELISA Kit (VAL609, Novus), Human IL‐6 ELISA Kit (QK206 R&D Systems) and Human TNF‐α ELISA Kit (NBP1‐91170, Novus).

### 2.4 Quantitative real‐time PCR

Total RNA was extracted from cells by RNAiso Plus (Takara). Each sample was reverse‐transcribed by PrimeScript™ RT Reagent Kit (Takara). The qPCR was performed by Lightcycler 480 System PCR System with SYBR Premix Ex Taq II. PCR procedure contained pre‐denaturation at 95°C for 30 s, followed by 40 cycles of denaturation at 95°C for 5 s, annealing at 60°C for 30 s, and extension at 72°C for 30 s, and finalized with melting at 95°C for 30 s. Sequences of mouse and human *NRF2*, *GCLC* and *NQO1* mRNA primers were available in the previous studies.^42^, ^43^ Other gene primers were designed using Primer Premier (V6.1) and synthesized by Biotune Biotechnology. The primer sequences were given in Table 1.

**TABLE 1:** Primer sequences used for qPCR. ‘F’ and ‘R’ indicate forward and reverse primers respectively

Genes	Primer sequences
Mouse *Il*‐*6*	F: 5′‐GCCTCTCTCTGACATGCT‐3′
	R: 5′‐GCCATTGCATTCTCTCTCTCTGCT‐3′
Mouse *Mcp*‐*1*	F: 5′‐CTGGACCCATGCATGCTT‐3′
	R: 5′‐CTGGCTCTCTCTCTTTCTTC‐3′
Mouse *Nrf2*	F: 5′‐TGCCTCCAAAGGATGTCAAT‐3′
	R: 5′‐CCTCTGCTGCAAGTAGCCTC‐3′
Mouse *Gclc*	F: 5′‐GTCTCAAGAACATCGCCTCC‐3′
	R: 5′‐CTGCACATCTACCACGCAGT‐3′
Mouse *Nqo1*	F: 5′‐AATGGGCCAGTACAATCAGG‐3′
	R: 5′‐CCAGCCCTAAGGATCTCTCC‐3′
Mouse *Gapdh*	F: 5′‐TGTCTCCTGCGACTTCAACA‐3′
	R: 5′‐GGTGGTCCAGGGTTTCTTACT‐3′
Human *IL*‐*6*	F: 5′‐TGCAATAACCCCTGACC‐3′
	R: 5′‐ATTTGCCGAAGCCCG‐3′
Human *TNF*‐*α*	F: 5′‐GAGCAAGCCCTGGTATG‐3′
	R: 5′‐CGGGCCGATGAGATGATCTCTCG‐3′
Human *NRF2*	F: 5′‐TACTCCCAGGTTGCCCACA‐3′
	R: 5′‐CATCTACAAACGGGAATGTCTGC‐3′
Human *GCLC*	F: 5′‐TCTCTAATAAAGAGATGAGCAACATGC‐3′
	R: 5′‐TTGACGATAGATAAAGAGATCTACGAA‐3′
Human *NQO1*	F: 5′‐CCTGGAAGGATGGAAGAAAG‐3′
	R: 5′‐AGAATCCTGCCTGGAAGTTTAGC‐3′
Human *GAPDH*	F: 5′‐GCACCGTCAAGGCTGAGAAC‐3′
	R: 5′‐TGGTGAAGACGCCAGTGGA‐3′

### 2.5 NF‐κB p65 transcription factor assay

The NF‐κB p65 transcription factor assay was conducted using the NF‐κB p65 TF Assay Kit based on the manufacturer's instructions. Nuclear lysate was extracted by Nuclear Protein Extraction Kit. The nuclear lysate of 10 μl was placed in a 96‐well plate to react with the dsDNA. After multiple cleanings, 100 μl primary antibody of NF‐κB (kept at room temperature for 2 h) and 100 μl goat anti‐rabbit horseradish peroxidase (HRP) conjugate antibody (kept at room temperature for 2 h) were added into the reaction well in turn. After one‐step colour reaction, the absorbance at 450 nm was observed and the OD value was calculated.

### 2.6 Arthritis model in vivo

The male C57BL6 mice were purchased from Charles River. After anaesthesia, the medial meniscus ligament was cut‐off with a microblade, dissociated, with the tissue and skin sutured layer by layer to establish a surgical destabilization of the medial meniscus model of osteoarthritis (DMM). LPS was used to enhance the development of OA in mice. Specifically, the mice were injected with LPS (2.5 mg/kg) into the knee joint, and the control group was injected with the same volume of normal saline solution at the age of 8 weeks. Then, OI (50 mg/kg) was injected into the knee joint three times in a week on the 1st, 4th and 7th day. All the mice were treated with the left hindlimb to keep the mice free to move.

### 2.7 **Nrf2** **knockout in a mouse arthritis model**

To establish a mouse arthritis model. LPS (2.5 mg/kg) was used to enhance the development of OA in mice. Then, OI (50 mg/kg) was injected into the knee joint three times in a week on the 1st, 4th and 7th day, and ML385 was injected intraperitoneally 1 h before each injection. All the mice were treated with the left hindlimb to keep the mice free to move.

### 2.8 Haematoxylin‐eosin (HE) staining

The distal femur was dissected and fixed with 4% PFA and decalcified. Paraffin sections were quickly stained by HE with the cytoplasm stained red and the nucleus stained blue.

### 2.9 Safranin O‐Fast green staining

The distal femur was dissected and fixed with 4% PFA and decalcified. Paraffin sections were quickly stained by safranin O‐fast green. The cartilage matrix was stained dark red, and the cytoplasm, muscle, collagen fibre and bone tissue were stained green to directly reflect the structure of articular cartilage and bone tissue.

### 2.10 OARSI Scoring

The histology of articular cartilage in mice was graded based on the Osteoarthritis Research Society International (OARSI) score system. The score criteria included the absence/presence of safranin O staining, the fracture depth of articular cartilage and the cumulative degree of articular surface.

### 2.11 Cell viability assay

The mouse ATDC5 cells and human C28I2 cells were seeded in 96‐well culture plates. Once the density of the cells treated with LPS and OI reached 70%, CCK‐8 (10 μl) was added to stain for 30 min. Then, the absorbance at 550 nm was observed, and the OD value was calculated.

### 2.12 LDH cytotoxicity assay

The mouse ATDC5 cells and human C28I2 cells were inoculated in 96‐well culture plates. When the density of cells treated with LPS and OI reached 70%, the supernatant (120 μl) was collected to react with 60 μl LDH working solution for 2 h. Then, the absorbance was observed at 490 nm, and OD value was calculated.

### 2.13 Trypan Blue staining cell viability assay

The mouse ATDC5 cells and human C28I2 cells were cultured in 6‐well culture plates at a density of 1 × 10^6^ cells/well. After the treatment of LPS and OI, cells were suspended by EDTA‐free trypsin and added with equal volume of diluted 1× Trypan Blue stain. The blue ‘dead’ cells were counted using a blood cell count plate, and the ratio (100%) of ‘dead’ cells to the total cells was calculated.

### 2.14 Flow cytometry

The mouse ATDC5 cells and human C28I2 cells were cultured in 6‐well culture plates at a density of 1 × 10^6^ cells/well. After the treatment of LPS and OI, cells were suspended by EDTA‐free trypsin with the cells in the supernatant also collected. Each sample was incubated with 5 μl PE Annexin V and 7‐ADD. The excitation voltage was adjusted according to the cell size. The number of apoptotic cells in 1 × 10^5^ cells was counted by fluorescent‐activated cell sorting (FACS).

### 2.15 TUNEL assay

The mouse ATDC5 cells and human C28I2 cells seeded in 96‐well culture plates at 0.5 × 10^5^ cells/well were permeabilized with proteinase K (20 μg/ml) at room temperature for 5 min. Each sample was stained with 50 μl TUNEL TdT Enzyme working solution for 30 min. The fluorescence intensity of the sample was detected with EX at 546 nm and EM at 570 nm.

### 2.16 Western blotting assay

The mouse ATDC5 cells and human C28I2 cells were cultured in 100 mm Cell Culture Dishes with a density of 2 × 10^6^ cells/dish. After the treatment of OI, the cells were collected by centrifugation. Cytoplasmic protein lysate and nucleoprotein lysate were successively added with PMSF. The protein sample (20 μg/lane) was evaluated with electrophoresis and transfected into 0.22 μm PVDF which was stained with ECL after incubating with the primary antibody at 1:1000 dilution at 4°C overnight and goat anti‐rabbit secondary antibody at 1:5000 dilution at 25°C for 1.5 h. The total grey scale of each strip was quantified by ImageJ software with the values normalized based on the house‐keeping proteins (i.e., β‐actin and Lamin B1).

### 2.17 Immunofluorescence

Based on a previous study, the cells were immobilized with 4% PFA for 30 min and sealed with 5% BSA containing 0.1% Triton‐X for 1 h.^44^ The cells were incubated with primary antibody at 4°C overnight and then incubated with secondary antibody at 25°C for 1 h. The protein expression and localization performed in dark were observed under the inverted laser scanning confocal microscope.

### 2.18 Nqo1 activity assay

As previously described, a total of 100 μl menadione was used as substrate to react with the lysate for 48 h.^19^ The activity of Nqo1 was assessed in cell lysates. The OD value of the mixture was calculated at 565 nm.

### 2.19 Nrf2 siRNA

Based on the *Nrf2* sequence, the Nrf2 siRNAs were designed and synthesized by Genepharma (Table 2). The same amount of Lipofectamine‐3000 and si‐Nrf2 (5 μl) were premixed in 200 μl opti‐MEM. The cells were treated with si‐Nrf2 working solution for 24–48 h. The knockout efficiency was evaluated by Wb and qPCR.

**TABLE 2:** si‐Nrf2 sequences used in this study. ‘F’ and ‘R’ indicate forward and reverse primers respectively

si‐RNAs	Primer sequences
Mouse si‐306	F: 5′‐GGAUUUGAUUGACAUCCUUTT‐3′
	R: 5′‐AAGGAUGUCAAUCAAAUCCTT‐3′
Mouse si‐815	F: 5′‐CCGAAUUACAGUGUCUUAATT‐3′
	R: 5′‐UUAAGACACUGUAAUUCGGTT‐3′
Mouse si‐2021	F: 5′‐GCAAGAAGCCAGAUACAAATT‐3′
	R: 5′‐UUUGUAUCUGGCUUCUUGCTT‐3′
Human si‐772	F: 5′‐GGUUGAGACUACCAUGGUUTT‐3′
	R: 5′‐AACCAUGGUAGUCUCAACCTT‐3′
Human si‐811	F: 5′‐GACAGAAGUUGACAAUUAUTT‐3′
	R: 5′‐AUAAUUGUCAACUUCUGUCTT‐3′
Human si‐1220	F: 5′‐CCAGAACACUCAGUGGAAUTT‐3′
	R: 5′‐AUUCCACUGAGUGUUCUGGTT‐3′

### 2.20 Statistical analysis

Each experiment with 3 or 4 samples included was repeated thrice. Data presented as the mean ± standard deviation were subjected to one‐way ANOVA (nonparametric or mixed) and two‐tailed unpaired *t*‐test by Graphpad Prism (V8.4.0). The significant level was set at *p* < 0.05.

## 3 RESULTS

### 3.1 OI inhibits the inflammation induced by LPS in mouse ATDC5 cells and human C28I2 cells

**FIGURE 1:**
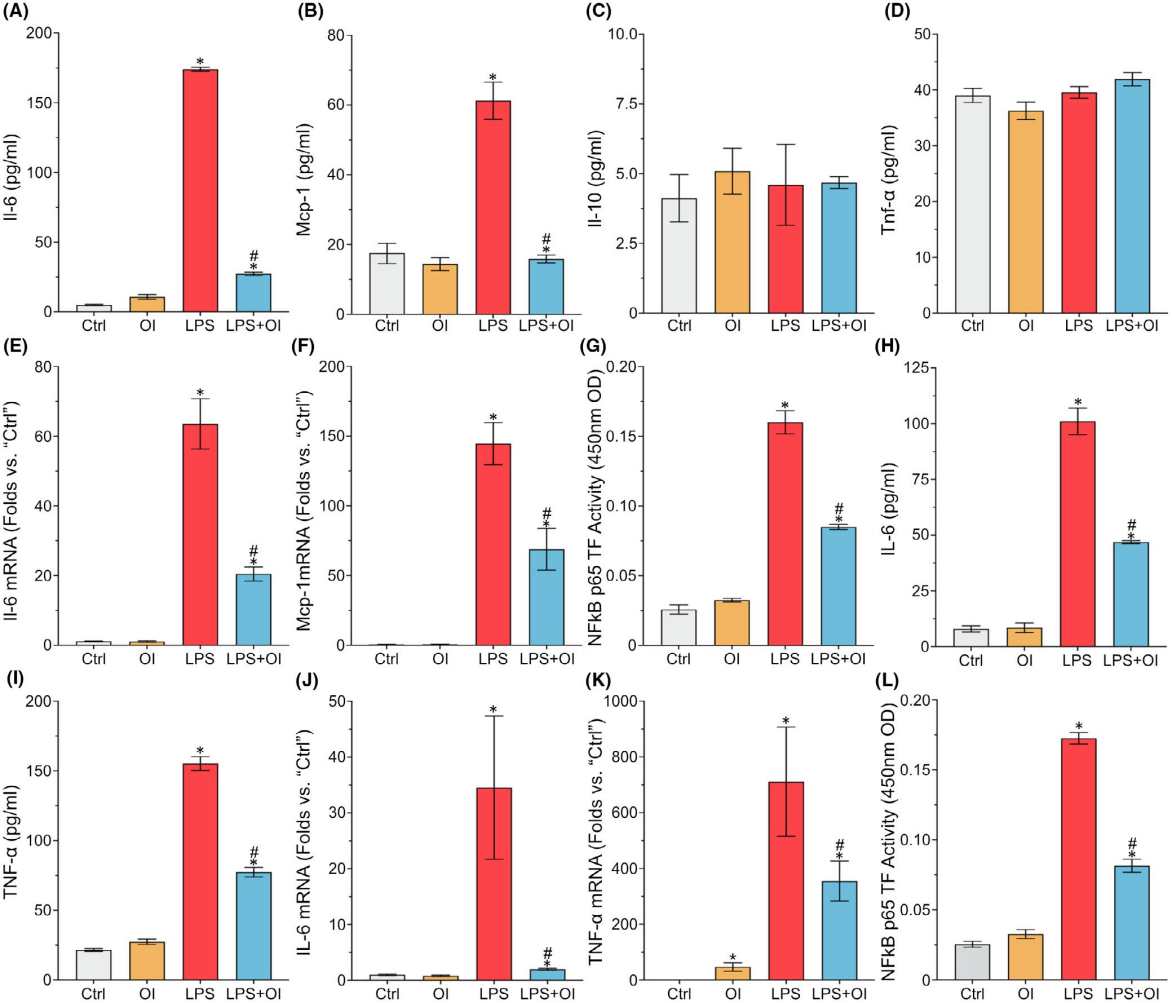
4‐octyl itaconate inhibits the inflammatory reaction in mouse ATDC5 cells and human C28I2 cells. The ATDC5 cells and C28I2 cells are induced with LPS (10 μg/ml) for 12 h and then treated with OI (100 μm) for 12 h. The protein levels of Il‐6, MCP‐1, Il‐10 and Tnf‐α are detected by ELISA (A–D, H, and I). The mRNA in cells is detected by qPCR (E, F, J and K). The NF‐κB p65 transcriptional activity in nucleus is detected by the NF‐κB p65 Transcription Factor Assay Kit (G and L). Each experiment is repeated three times. Data are presented as mean ± standard deviation (*n *= 3). Symbols ‘*’and ‘#’ indicate the significant difference set at *p* < 0.05 in comparison with control (‘Ctrl’) and LPS induction respectively

To investigate the potential effects of OI on pro‐inflammatory cytokines, the mouse ATDC5 cells were first induced with LPS (10 μg/ml) for 12 h. Compared with the control group, the levels of Il‐6 and Mcp‐1 were significantly increased with the LPS induction (Figure 1A,B), whereas no significant change was revealed in the expression levels of Il‐10 and Tnf‐α (Figure 1C,D). With the treatment of OI, the levels of Il‐6 and Mcp‐1 in the supernatant were notably inhibited (Figure 1A,B). The levels of IL‐10 and TNF‐α exhibited a low sensitivity to OI (Figure 1C,D). LPS‐induced production of *Il*‐*6* and *Mcp*‐*1* mRNAs was also enormously inhibited by OI treatment (Figure 1E,F). The treatment of OI significantly reduced the nuclear transcription activity of NF‐κB p65 in ATDC5 cells (Figure 1G).

High levels of IL‐6 and TNF‐α were revealed in the human C28I2 cells with LPS induction. Treatment with OI significantly repressed the production of IL‐6 and TNF‐α (Figure 1H,I). A similar increasing pattern was observed in the expression of both *IL*‐*6* and *TNF*‐*α* at mRNA level in the human C28I2 cells (Figure 1J,K). Furthermore, OI significantly reduced NF‐κB P65 transcription activity in the human C28I2 cells (Figure 1L). Our results evidently showed that OI effectively inhibited the production of pro‐inflammatory factors in both ATDC5 and C28I2 cells treated with LPS.

### 3.2 OI mitigates inflammatory injury of articular cartilage in a mouse arthritis model

**FIGURE 2:**
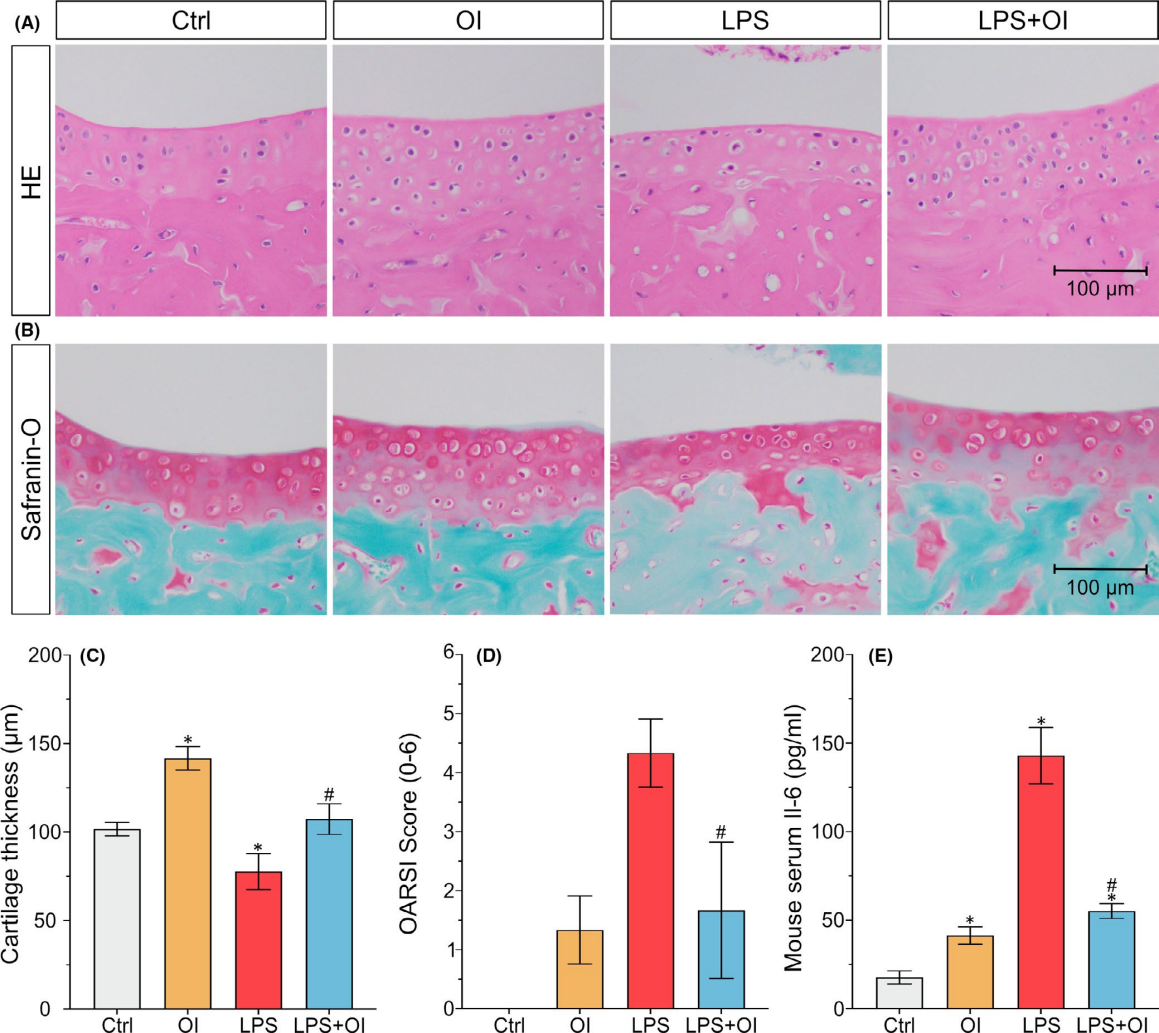
4‐octyl itaconate inhibits the inflammatory injury in C57BL6 mice. The distal femurs of mice are sectioned with the degree of cartilage injury detected by HE staining (A) and Safranin‐O‐fast green staining (B) and the thickness of cartilage measured (C). Comparisons in OARSI cartilage damage are scored based on the degree of cartilage injury (D). The serum of mice is extracted with the content of IL‐6 in serum detected by ELISA (E). Each experiment is repeated three times. Data are presented as mean ± standard deviation (*n* = 3). Symbols ‘*’and ‘#’ indicate the significant difference set at *p* < 0.05 in comparison with control (‘Ctrl’) and LPS induction respectively

A surgical mouse model of OA was established to study the protective effect of OI on inflammatory injury of OA cartilage in vivo. Results of the histological investigations based on both HE staining and safranin‐O staining showed that injection of LPS after DMM operation induced inflammatory injury of articular cartilage. Compared with the control group, the number of articular chondrocytes and the thickness of cartilage layer decreased in the LPS injection group (Figure 2A–C). Conversely, the density of chondrocytes and the thickness of cartilage layer were significantly improved in OI treatment group (Figure 2A–C). Furthermore, the OARSI scores were consistent with the results derived from both HE staining and safranin‐O staining. The OARSI scores were decreased after OI treatment (Figure 2D). The level of Il‐6 in serum was detected in order to verify the systemic effect of LPS injection into knee joint. The results showed that OI reduced the level of Il‐6 in vivo of OA induced by LPS (Figure 2E). These results demonstrated that OI alleviated the inflammatory damage caused by LPS in DMM mice.

### 3.3 OI attenuates cell death and apoptosis in mouse ATDC5 cells and human C28I2 cells induced by LPS

**FIGURE 3:**
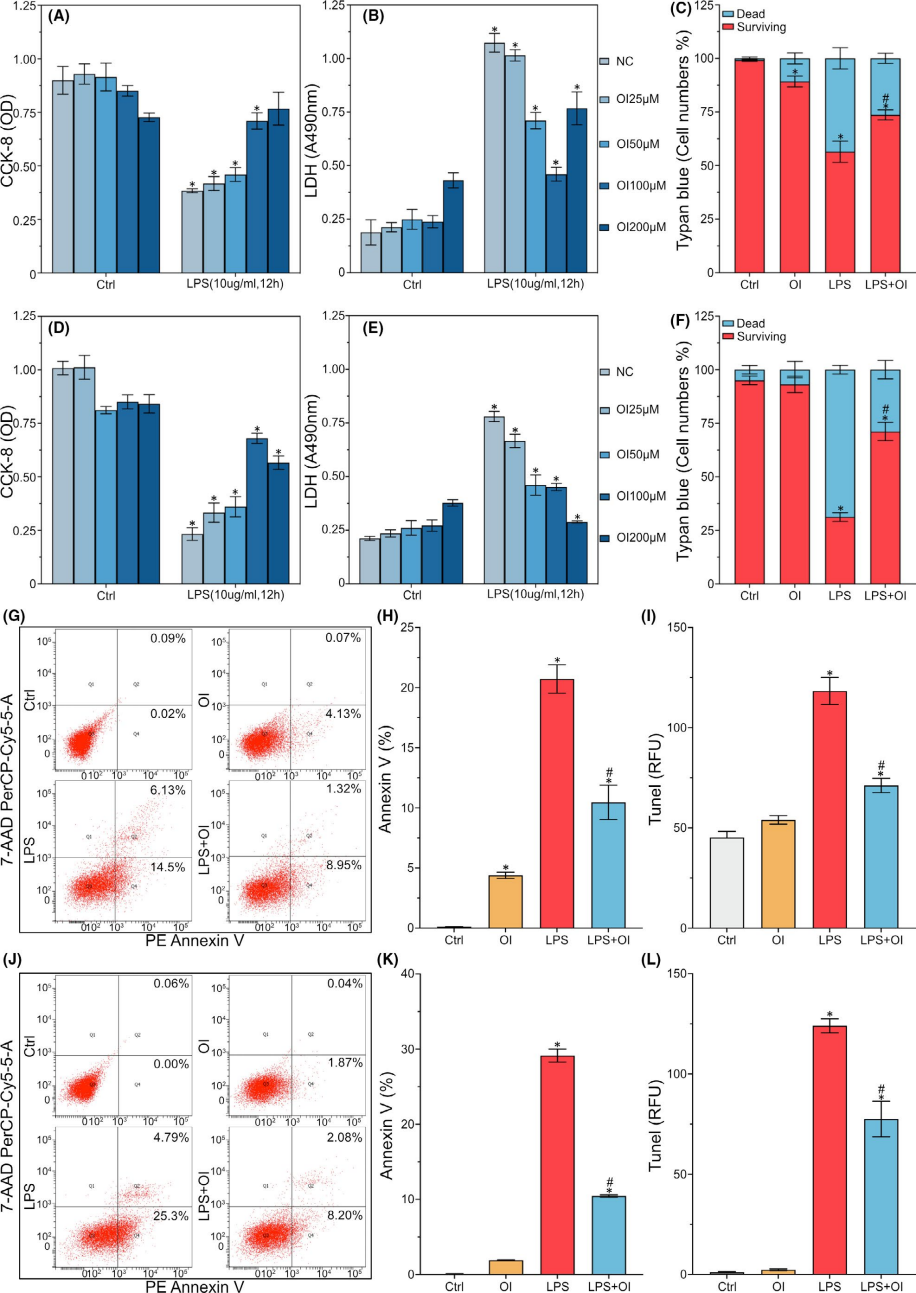
Cells necrosis and apoptosis in LPS‐induced inflammatory reaction in mouse ATDC5 cells and human C28I2 cells. The ATDC5 cells and the C28I2 cells are stimulated with LPS (10 μg/ml) for 12 h, followed immediately by treatment of OI with different concentrations (25, 50, 100 and 200 μM) for 12 h. The cell viability and LDH release level are tested by CCK‐8 Kit and LDH Cytotoxity Assay Kit (A, B, D and E). The dead cell count is evaluated by Trypan Blue straining assay (C and F). PE Annexin V is tested by PE Annexin V Apoptosis Detection Kit (G, H, J and K). TUNEL staining ratio is detected by the One Step TUNEL Apoptosis Assay Kit (I and L). Data are presented as mean ± standard deviation (*n* = 3). Symbols ‘*’ and ‘#’ indicate the significant difference set at *p* < 0.05 in comparison with control (‘Ctrl’) and LPS induction respectively

Results showed that the mouse ATDC5 cells treated with LPS (10 μg/ml, 12 h) showed decreased cell viability (Figure 3A), increased LDH release level (Figure 3B) and induced cell death (Figure 3C). Treatment of OI significantly inhibited LPS‐induced cytotoxicity in the ATDC5 cells in a dose‐dependent manner (Figure 3A,B). A single treatment of OI at 25, 50, 100 and 200 μM revealed no significant difference in cytotoxicity (Figure 3A,B). OI was also demonstrated to show the ability to reduce the damage to LPS‐induced cell viability (Figure 3D), increase LDH release levels (Figure 3E) and induce cell death (Figure 3F) in the human C28I2 cells. The results showed that OI (100 μM) efficiently protected the ATDC5 and C28I2 cells from the injury induced by LPS (Figure 3A,B,D,E). Therefore, the 100 μM was chosen as the optimal concentration of OI for further experiments.

The potential effect of OI on apoptosis was further verified by flow cytometry analysis. Results showed that the ratio of apoptosis and death rate induced by LPS was significantly increased, while the ratio of Annexin V was decreased in the ATDC5 cells treated with OI (Figure 3G,H). The TUNEL staining ratio was also decreased in the ATDC5 cells treated with OI (Figure 3I). The apoptosis activation was evidently induced by LPS as demonstrated by increased Annexin V ratio (Figure 3J,K) and TUNEL staining (Figure 3L). These results showed that OI played an important role in the protection of chondrocytes.

### 3.4 OI activates Nrf2 signalling pathway and downstream genes in mouse ATDC5 cells and human C28I2 cells

**FIGURE 4:**
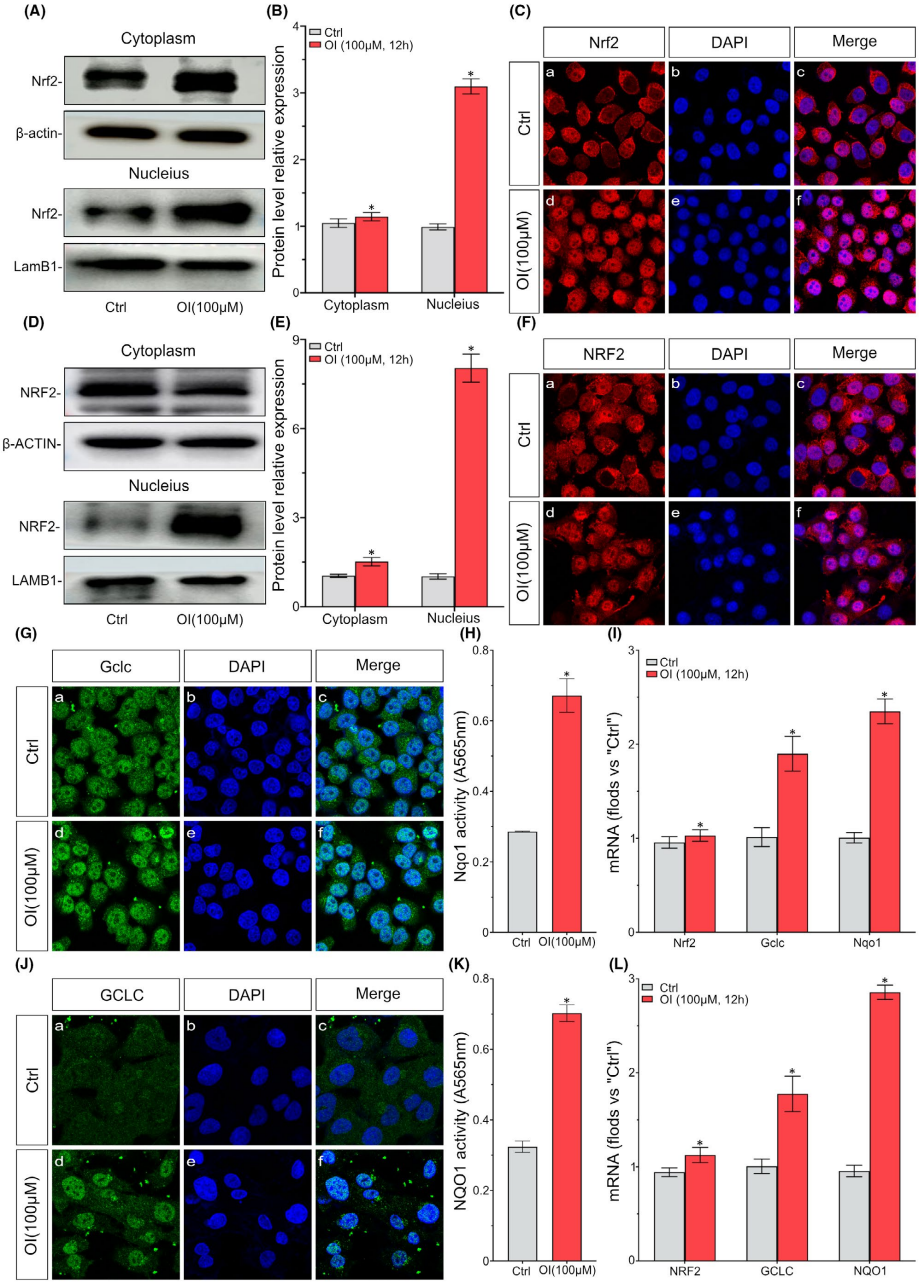
4‐octyl itaconate activates Nrf2 signalling pathway and downstream genes in mouse ATDC5 cells and human C28I2 cells. The ATDC5 cells and the C28I2 cells are treated with OI (100 μM) for 12 h. The expression of Nrf2 protein in cytoplasm and nucleus is analysed by Western blot (A, B, D and E). The protein expression and localization are detected by IF (C and F). The protein expression and localization of Gclc are detected by IF (G and J). Using menadione as substrate, the Nqo1 activity of lysates is determined by ELISA (H and K). The mRNA levels of Nrf2, Gclc and Nqo1 in cells are detected by qPCR (I and L). Data are presented as mean ± standard deviation (*n* = 3). Symbols ‘*’ indicates the significant difference set at *p *< 0.05 in comparison with control (‘Ctrl’)

The potential effects of OI on Nrf2 signalling pathway in ATDC5 cells were investigated. The expression of Nrf2 protein in cytoplasm and nucleus was detected after the stimulation of OI (100 μM, 12 h). The results revealed that the expression of Nrf2 protein was slightly increased in cytoplasm but significantly increased in nucleus (Figure 4A,B). The results of immunofluorescense analysis confirmed that Nrf2 was strongly expressed and accumulated in the nucleus under the OI treatment (Figure 4C). The results of the expression and localization analyses of NRF2 protein in C28I2 cells further showed that NRF2 was stabilized with enhanced nuclear translocation under the treatment of OI (Figure 4D–F), which was the crucial step of NRF2 activation.

The effects of OI on Nrf2 downstream gene expression were further verified by IF and activity analyses. Results showed that the expression of Gclc protein was significantly increased in ATDC5 cells treated with OI (Figure 4G) and the activity of Nqo1 was significantly increased (Figure 4H). The transcription levels of the Nrf2‐dependent genes *Gclc* and *Nqo1* were significantly increased in ATDC5 cells (Figure 4I). However, the overexpression of *Nrf2* mRNA was not achieved by the treatment of OI (Figure 4I). The Nrf2‐dependent molecule GCLC expression and NQO1 activity were significantly increased in C28I2 cells treated with OI (Figure 4J,K). The transcriptions of genes *GCLC* and *NQO1* were also enhanced in C28I2 cells (Figure 4L). Our results showed that OI stimulated Nrf2 signalling pathway in both the ATDC5 and C28I2 cells and promoted downstream genes expression.

### 3.5 Nrf2 activation involved in inhibiting LPS‐induced pro‐inflammatory reactions in mouse ATDC5 cells and human C28I2 cells treated with OI

**FIGURE 5:**
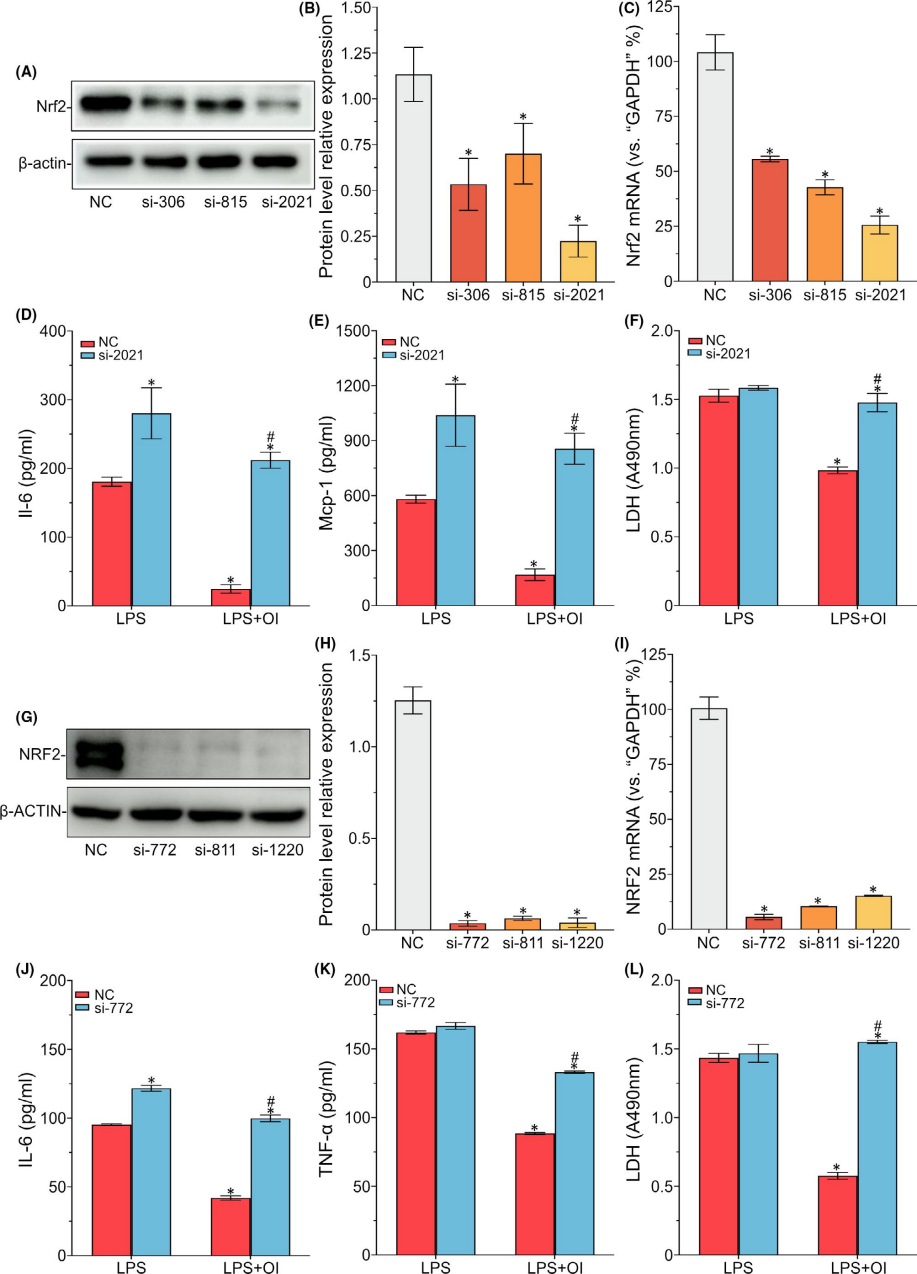
Nuclear factor erythroid‐2‐related factor 2 activation involved in the inhibition of LPS‐induced mouse ATDC5 cells and human C28I2 cells. The ATDC5 and C28I2 cells are pre‐transfected by the si‐RNAs and treated with OI (100 μM) for 12 h. The protein expression and mRNA of Nrf2 are detected by Wb and qPCR (A–C and G–I). The protein expression of Il‐6, Mcp‐1 and Tnf‐α in the supernatant of cells is detected by ELISA (D, E, J and K). The LDH release level is tested by LDH Cytotoxity Assay Kit (F and L). Symbols ‘*’ indicate the significant difference set at *p* < 0.05 in comparison with negative control (‘NC’) under LPS induction. Symbols ‘#’ indicate the significant difference set at *p* < 0.05 in comparison with negative control (‘NC’) under LPS and OI exposure

The interference of OI on LPS‐induced ATDC5 and C28I2 cells was investigated through the positive regulation of Nrf2 signalling pathway. A series of Nrf2 si‐RNAs were used to knockout Nrf2 in the ATDC5 cells. The knockout efficiency of si‐RNAs was measured at both protein and mRNA levels. Compared with the control group, the Nrf2 level of ATDC5 cells transfected with si‐2021 was dramatically decreased (Figure 5A–C). Therefore, si‐2021 was chosen as highly effective distractor in the subsequent experiments. After Nrf2 signalling pathway was inhibited in the ATDC5 cells, OI treatment did not restrain the LPS‐induced expression of Il‐6 and Mcp‐1 (Figure 5D,E) and LDH release level (Figure 5F). The si‐722 was used to efficiently knockout NRF2 in the C28I2 cells (Figure 5G–I), reversing the inhibitory effect of OI on LPS‐induced expression of IL‐6 and TNF‐α (Figure 5J,K) and LDH release level (Figure 5L). Upon the interference of Nrf2 signalling pathway, the protective effect of OI on chondrocytes was not observed. These results showed that the activation of Nrf2 mediated the inhibition of LPS‐induced inflammatory responses in the ATDC5 and C28I2 cells treated with OI.

### 3.6 The Nrf2 signalling pathway as the target for OI in a mouse arthritis model.

**FIGURE 6:**
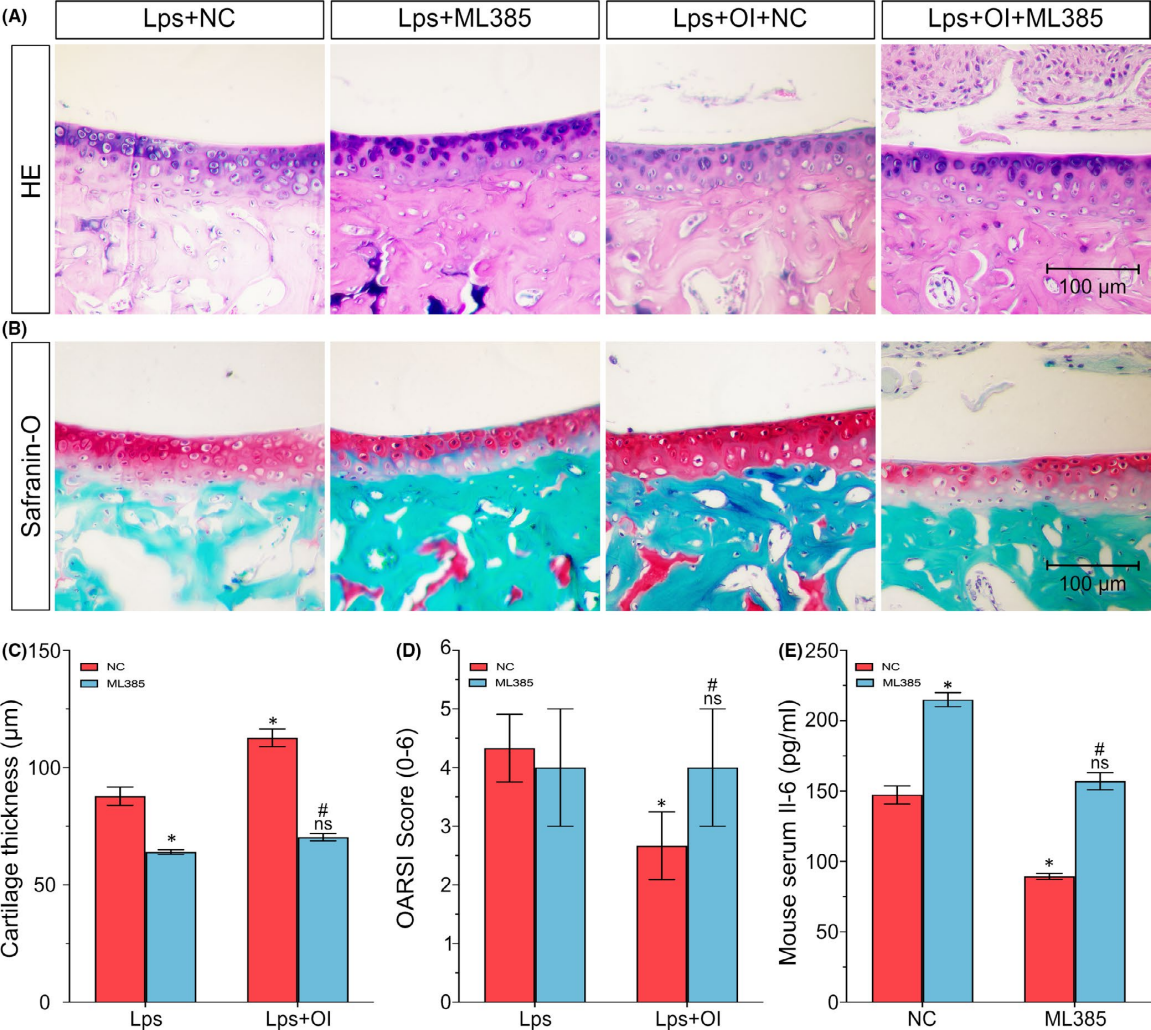
Nuclear factor erythroid‐2‐related factor 2 signalling pathway as the target for OI in a mouse arthritis model. The distal femurs of mice are sectioned with the degree of cartilage injury detected by HE staining (A) and Safranin‐O‐fast green staining (B) and the thickness of cartilage measured (C). Comparisons in OARSI cartilage damage are scored based on the degree of cartilage injury (D). The serum of mice is extracted with the content of IL‐6 in serum detected by ELISA (E). Each experiment is repeated three times. Data are presented as mean ± standard deviation (*n* = 3). Symbols ‘*’ indicate the significant difference set at *p* < 0.05 in comparison with negative control (‘NC’) under LPS induction. Symbols ‘ns’ indicate the insignificant difference set at *p* > 0.05 in comparison with negative control (‘NC’) under LPS induction. Symbols ‘#’ indicate the significant difference set at *p* < 0.05 in comparison with negative control (‘NC’) under LPS and OI exposure

To investigate the possible mechanism of OI in protecting inflammatory injury of arthritis in vivo. First, a surgical mouse model of OA was constructed, and the Nrf2 signalling pathway was blocked by intraperitoneal injection of ML385 (Nrf2 inhibitor). Results of the histological investigations based on both HE staining and safranin‐O staining showed that after the Nrf2 signal was blocked, the protective effect of OI on inflammatory injury of OA was weakened. Compared with the NC group under LPS and OI exposure, the number of articular chondrocytes and the thickness of cartilage layer decreased and the OARSI scores increased in the ML385 group under LPS and OI exposure. However, compared with NC group under LPS induce, there was no significant difference in ML385 under LPS and OI exposure (Figure 6A–D). Furthermore, the changes in serum Il‐6 levels are consistent with the above performance (Figure 6E). These results revealed that OI relieved cartilage injury by activating the Nrf2 signalling pathway in a mouse arthritis model.

## 4 DISCUSSION

Osteoarthritis affects 4%~7% of the population worldwide, causing severe clinical challenges and financial burdens for patients.^45^ Therefore, it is urgent to establish effective medical treatments on articular cartilage injury. Traditionally, OA is considered as a degenerative change of articular cartilage without the participation of inflammatory factors. Recent studies have shown that metabolic inflammation and inflammatory factors play essential roles in the pathogenesis of OA.^46^ Caused by either traumatic factors or self‐degenerative factors, patients of OA generate the combination of damps and pattern recognition receptors (PRRs), leading to the imbalance of inflammation and apoptosis.^47^ Furthermore, LPS could specifically activate TLR‐4 signal in cells, causing a strong pro‐inflammatory response.^48^ In our study, we used LPS to induce the mouse ATDC5 cells and human C28I2 cells to simulate the inflammatory injury of OA cartilage in vitro. LPS was shown to significantly induce IL‐6, MCP‐1 and TNF‐α, activate NF‐κB transcription, reduce cell activity and induce apoptosis. The chondrocyte inflammation model induced by LPS was effectively applied to reflect the degree of OA cartilage injury. These results are consistent with those reported previously.^9^, ^49^, ^50^, ^51^ However, the overexpression of TNF‐α was not observed with the treatment of OI in LPS‐induced ATDC5 cells. These results are not consistent with those previously reported, probably due to the different treatment time of LPS and cell density at the time of sampling.^50^ Future studies are necessary to investigate these inconsistent results.

Among various therapeutic strategies for cartilage injury and OA, the enhancement of endogenous defence mechanism (i.e., Nrf2 signalling pathway) by ingesting pharmacological effects of small molecules is potentially a promising medical treatment.^52^, ^53^ Many activators of Nrf2 have been demonstrated to be effective anti‐inflammatory agents, showing severe adverse reactions and poor biological activities.^54^, ^55^ In recent years, a great amount of efforts has been devoted to finding new Nrf2 activators in order to effectively protect chondrocytes from inflammatory damage. Our study confirmed for the first time that itaconate, as a novel Nrf2 activator, can effectively protect chondrocytes from inflammatory injury. Our results showed that OI promoted the expression of Nrf2 and downstream genes *GCLC* and *NQO1* not by inducing *Nrf2* gene transcription but by activating inactivated Nrf2 in the cytoplasm. These results provide strong evidence to support the application of itaconate in the treatment of inflammation.^19^

Previous studies have shown that itaconate impairs SDH activity in cells but not as effectively as traditional SDH inhibitors (e.g., sodium malonate), suggesting that itaconate may play an anti‐inflammatory role through different mechanisms.^19^ Besides activating Nrf2, itaconate and its derivatives have also been shown to regulate the IkBz‐ATF3 inflammatory axis.^20^ Our results suggest that OI activates Nrf2 signalling pathway in chondrocytes. Specifically, OI induces stabilization of Nrf2 protein and nuclear transposition, leading to the expression of Nrf2 target genes *Gclc* and *Nqo1*. More importantly, once the Nrf2 signalling pathway is blocked, the protective effect of OI on chondrocytes is not observed. These results confirm that Nrf2 signalling activation mediates OI to inhibit LPS‐induced chondrocyte inflammation. We note that it is important to further explore the potential functional mechanism of Nrf2 in the inhibition of LPS‐induced inflammatory injury of articular cartilage and related target genes activated by OI as well as the possible anti‐inflammatory and protective functions of Nrf2.

**FIGURE 7:**
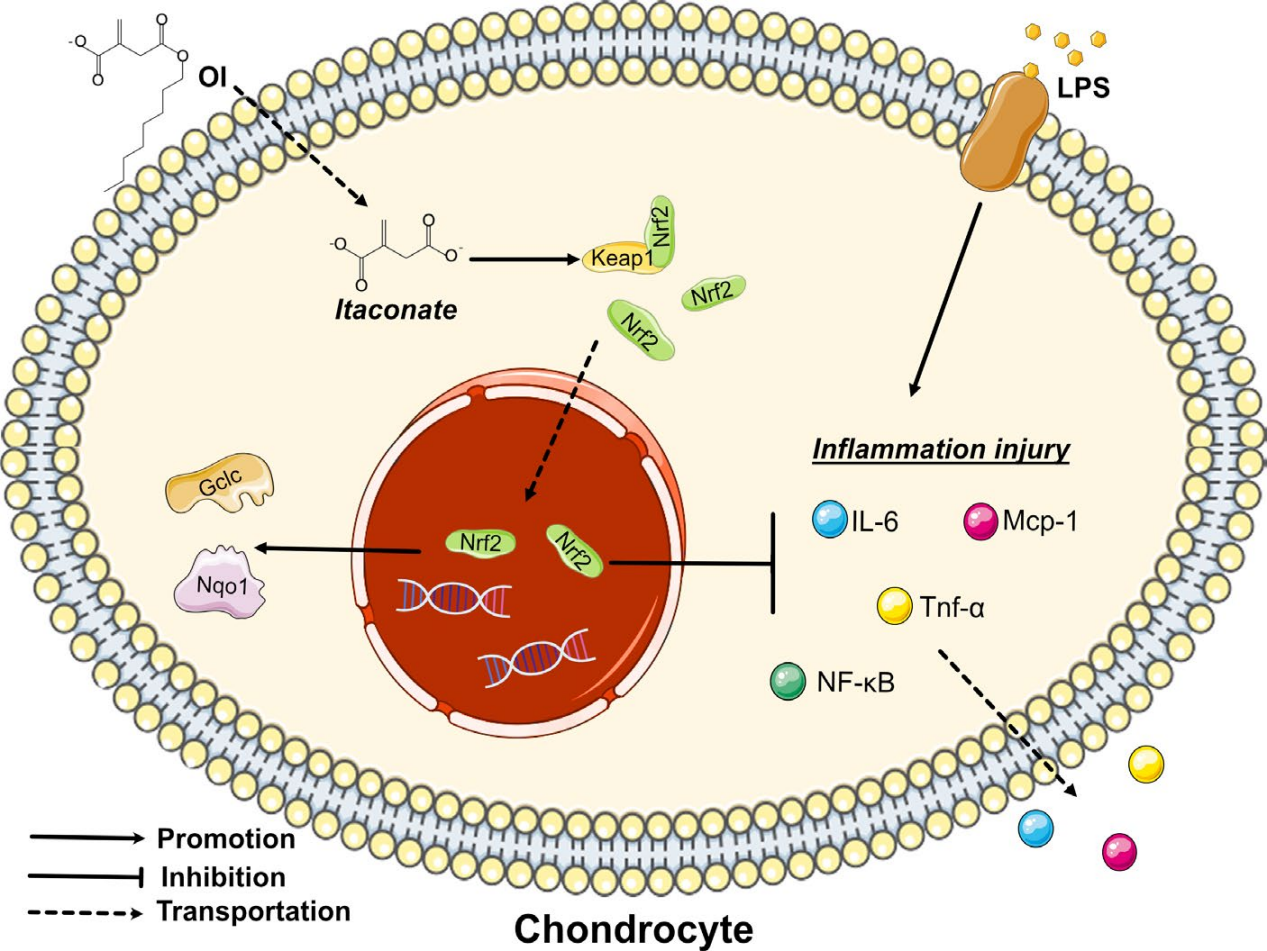
Schematic diagram of potential protective effects of OI on LPS‐induced inflammatory injury of chondrocytes

In conclusion, we report for the first time the effect and functional mechanism of itaconate in the treatment of cartilage inflammation both in vitro and in vivo. OI can effectively activate Nrf2 signalling pathway and downstream related anti‐inflammatory and anti‐apoptotic genes, ultimately inhibiting the production of LPS‐induced inflammatory factors, the NF‐κB activation, cell necrosis and apoptosis (Figure 7). Therefore, we conclude that itaconate contains significant importance for investigating the treatment of OA, opening a novel direction to explore other metabolites in the treatment of inflammation. More clinical trials are needed to verify in vivo the application of itaconate in the treatment of OA. We speculate that more in‐depth studies of metabolites in OA signalling pathway are necessary to make a breakthrough in the treatment of OA.

## CONFLICT OF INTEREST

The authors declare that there are no conflicts of interests.

## AUTHOR CONTRIBUTIONS

**Qingchen Zhang:** Data curation (lead); Methodology (lead); Writing – original draft (lead). **Xiaohui Bai:** Data curation (equal); Methodology (equal); Writing – review & editing (lead). **Rongrong Wang:** Methodology (supporting); Writing – original draft (supporting). **Hao Zhao:** Formal analysis (supporting); Methodology (supporting). **Lili Wang:** Methodology (supporting). **Jingwen Liu:** Formal analysis (supporting); Methodology (supporting); Writing – original draft (supporting). **Ming Li:** Formal analysis (supporting); Writing – review & editing (supporting). **Zheng Chen:** Formal analysis (supporting); Writing – review & editing (supporting). **Dawei Wang:** Conceptualization (lead); Project administration (lead); Supervision (lead); Writing – original draft (lead). **Zejun Wang:** Resources (lead). **Lianxin Li:** Formal analysis (lead); Validation (lead).

## Data Availability

The data sets used and/or analysed in this study can be obtained from the corresponding author on reasonable requirements.

## References

[1] Woolf AD , Pfleger B . Burden of major musculoskeletal conditions. Bull World Health Organ. 2003;81(9):646‐656. 14710506 PMC2572542

[2] Richard D , Liu Z , Cao J , et al. Evolutionary Selection and constraint on human knee chondrocyte regulation impacts osteoarthritis risk. Cell. 2020;181(2):362‐381.e28. 32220312 10.1016/j.cell.2020.02.057 PMC7179902

[3] Ji ML , Jiang H , Wu F , et al. Precise targeting of miR‐141/200c cluster in chondrocytes attenuates osteoarthritis development. Ann Rheum Dis. 2021;80(3):356‐366. 10.1136/annrheumdis-2020-218469 33109602

[4] Glyn‐Jones S , Palmer AJ , Agricola R , et al. Osteoarthritis. Lancet. 2015;386(9991):376‐387. 25748615 10.1016/S0140-6736(14)60802-3

[5] Carr AJ , Robertsson O , Graves S , et al. Knee replacement. Lancet. 2012;379(9823):1331‐1340. 22398175 10.1016/S0140-6736(11)60752-6

[6] Bijlsma JW , Berenbaum F , Lafeber FP . Osteoarthritis: an update with relevance for clinical practice. Lancet. 2011;377(9783):2115‐2126. 21684382 10.1016/S0140-6736(11)60243-2

[7] Scanzello CR . Chemokines and inflammation in osteoarthritis: insights from patients and animal models. J Orthop Res. 2017;35(4):735‐739. 27808445 10.1002/jor.23471 PMC5912941

[8] Woodell‐May JE , Sommerfeld SD . Role of inflammation and the immune system in the progression of osteoarthritis. J Orthop Res. 2020;38(2):253‐257. 31469192 10.1002/jor.24457

[9] Zhao Z , Dai XS , Wang ZY , Bao ZQ , Guan JZ . MicroRNA‐26a reduces synovial inflammation and cartilage injury in osteoarthritis of knee joints through impairing the NF‐κB signaling pathway. Biosci Rep 2019;39(4):BSR20182025. 30872407 10.1042/BSR20182025 PMC6454017

[10] Brestoff JR , Artis D . Commensal bacteria at the interface of host metabolism and the immune system. Nat Immunol. 2013;14(7):676‐684. 23778795 10.1038/ni.2640 PMC4013146

[11] Santos LC , de Moraes AN , Saito ME . Effects of intraarticular ropivacaine and morphine on lipopolysaccharide‐induced synovitis in horses. Vet Anaesth Analg. 2009;36(3):280‐286. 19397780 10.1111/j.1467-2995.2009.00452.x

[12] Mendez ME , Sebastian A , Murugesh DK , et al. LPS‐induced inflammation prior to injury exacerbates the development of post‐traumatic osteoarthritis in mice. J Bone Miner Res. 2020;35(11):2229‐2241. 32564401 10.1002/jbmr.4117 PMC7689775

[13] Zhou Y , Chen X , Qu N , Zhang B , Xia C . Chondroprotection of PPARα activation by WY14643 via autophagy involving Akt and ERK in LPS‐treated mouse chondrocytes and osteoarthritis model. J Cell Mol Med. 2019;23(4):2782‐2793. 30729704 10.1111/jcmm.14184 PMC6433667

[14] Li F , Xu W , Zhao S . Regulatory roles of metabolites in cell signaling networks. J Genet Genomics. 2013;40(7):367‐374. 23876777 10.1016/j.jgg.2013.05.002

[15] Murphy MP , O'Neill LAJ . Krebs cycle reimagined: the emerging roles of succinate and itaconate as signal transducers. Cell. 2018;174(4):780‐784. 30096309 10.1016/j.cell.2018.07.030

[16] Michelucci A , Cordes T , Ghelfi J , et al. Immune‐responsive gene 1 protein links metabolism to immunity by catalyzing itaconic acid production. Proc Natl Acad Sci USA. 2013;110(19):7820‐7825. 23610393 10.1073/pnas.1218599110 PMC3651434

[17] Strelko CL , Lu W , Dufort FJ , et al. Itaconic acid is a mammalian metabolite induced during macrophage activation. J Am Chem Soc. 2011;133(41):16386‐16389. 21919507 10.1021/ja2070889 PMC3216473

[18] Li MH , Xiao R , Li JB , Zhu Q . Regenerative approaches for cartilage repair in the treatment of osteoarthritis. Osteoarthritis Cartilage. 2017;25(10):1577‐1587. 28705606 10.1016/j.joca.2017.07.004

[19] Mills EL , Ryan DG , Prag HA , et al. Itaconate is an anti‐inflammatory metabolite that activates Nrf2 via alkylation of KEAP1. Nature. 2018;556(7699):113‐117. 29590092 10.1038/nature25986 PMC6047741

[20] Bambouskova M , Gorvel L , Lampropoulou V , et al. Electrophilic properties of itaconate and derivatives regulate theIκBζ‐ATF3 inflammatory axis. Nature. 2018;556(7702):501‐504. 29670287 10.1038/s41586-018-0052-z PMC6037913

[21] Li R , Zhang P , Wang Y , Tao K . Itaconate: a metabolite regulates inflammation response and oxidative stress. Oxid Med Cell Longev. 2020;2020:5404780. 32724492 10.1155/2020/5404780 PMC7382747

[22] Williams NC , O’Neill LAJ . A role for the Krebs cycle intermediate citrate in metabolic reprogramming in innate immunity and inflammation. Front Immunol. 2018;9:141. 29459863 10.3389/fimmu.2018.00141 PMC5807345

[23] Perico L , Wyatt CM , Benigni A . A new BEACON of hope for the treatment of inflammation? The endogenous metabolite itaconate as an alternative activator of the KEAP1‐Nrf2 system. Kidney Int. 2018;94(4):646‐649. 30243306 10.1016/j.kint.2018.07.018

[24] Li W , Kong AN . Molecular mechanisms of Nrf2‐mediated antioxidant response. Mol Carcinog. 2009;48(2):91‐104. 18618599 10.1002/mc.20465 PMC2631094

[25] Suzuki T , Yamamoto M . Molecular basis of the Keap1‐Nrf2 system. Free Radic Biol Med. 2015;88(Pt B):93‐100. 26117331 10.1016/j.freeradbiomed.2015.06.006

[26] Itoh K , Tong KI , Yamamoto M . Molecular mechanism activating Nrf2‐Keap1 pathway in regulation of adaptive response to electrophiles. Free Radic Biol Med. 2004;36(10):1208‐1213. 15110385 10.1016/j.freeradbiomed.2004.02.075

[27] McMahon M , Lamont DJ , Beattie KA , Hayes JD . Keap1 perceives stress via three sensors for the endogenous signaling molecules nitric oxide, zinc, and alkenals. Proc Natl Acad Sci USA. 2010;107(44):18838‐18843. 20956331 10.1073/pnas.1007387107 PMC2973898

[28] Dinkova‐Kostova AT , Kostov RV , Canning P . Keap1, the cysteine‐based mammalian intracellular sensor for electrophiles and oxidants. Arch Biochem Biophys. 2017;617:84‐93. 27497696 10.1016/j.abb.2016.08.005 PMC5339396

[29] Wardyn JD , Ponsford AH , Sanderson CM . Dissecting molecular cross‐talk between Nrf2 and NF‐κB response pathways. Biochem Soc Trans. 2015;43(4):621‐626. 26551702 10.1042/BST20150014 PMC4613495

[30] Zhang Q , Pi J , Woods CG , Andersen ME . A systems biology perspective on Nrf2‐mediated antioxidant response. Toxicol Appl Pharmacol. 2010;244(1):84‐97. 19716833 10.1016/j.taap.2009.08.018 PMC2837757

[31] Bai XH , Wang DW , Luan Y , Yu XP , Liu CJ . Regulation of chondrocyte differentiation by ADAMTS‐12 metalloproteinase depends on its enzymatic activity. Cell Mol Life Sci. 2009;66(4):667‐680. 19151918 10.1007/s00018-008-8633-x PMC11131527

[32] Khan NM , Haseeb A , Ansari MY , Devarapalli P , Haynie S , Haqqi TM . Wogonin, a plant derived small molecule, exerts potent anti‐inflammatory and chondroprotective effects through the activation of ROS/ERK/Nrf2 signaling pathways in human Osteoarthritis chondrocytes. Free Radic Biol Med. 2017;106:288‐301. 28237856 10.1016/j.freeradbiomed.2017.02.041 PMC5490997

[33] Bolduc JA , Collins JA , Loeser RF . Reactive oxygen species, aging and articular cartilage homeostasis. Free Radic Biol Med. 2019;132:73‐82. 30176344 10.1016/j.freeradbiomed.2018.08.038 PMC6342625

[34] Cai D , Yin S , Yang J , Jiang Q , Cao W . Histone deacetylase inhibition activates Nrf2 and protects against osteoarthritis. Arthritis Res Ther. 2015;17:269. 26408027 10.1186/s13075-015-0774-3 PMC4583998

[35] Bai XH , Wang DW , Kong L , et al. ADAMTS‐7, a direct target of PTHrP, adversely regulates endochondral bone growth by associating with and inactivating GEP growth factor. Mol Cell Biol. 2009;29(15):4201‐4219. 19487464 10.1128/MCB.00056-09 PMC2715794

[36] Tang C , Wang X , Xie Y , et al. 4‐Octyl Itaconate Activates Nrf2 signaling to inhibit pro‐inflammatory cytokine production in peripheral blood mononuclear cells of systemic lupus erythematosus patients. Cell Physiol Biochem. 2018;51(2):979‐990. 30466076 10.1159/000495400

[37] Tang C , Tan S , Zhang Y , Dong L , Xu Y . Activation of Keap1‐Nrf2 signaling by 4‐octyl itaconate protects human umbilical vein endothelial cells from high glucose. Biochem Biophys Res Commun. 2019;508(3):921‐927. 30545629 10.1016/j.bbrc.2018.12.032

[38] Liu H , Feng Y , Xu M , Yang J , Wang Z , Di G . Four‐octyl itaconate activates Keap1‐Nrf2 signaling to protect neuronal cells from hydrogen peroxide. Cell Commun Signal. 2018;16(1):81. 30442144 10.1186/s12964-018-0294-2 PMC6238317

[39] Song H , Xu T , Feng X , et al. Itaconate prevents abdominal aortic aneurysm formation through inhibiting inflammation via activation of Nrf2. EBioMedicine. 2020;57:102832. 32574955 10.1016/j.ebiom.2020.102832 PMC7322255

[40] Sun X , Zhang B , Pan X , et al. Octyl itaconate inhibits osteoclastogenesis by suppressing Hrd1 and activating Nrf2 signaling. FASEB J. 2019;33(11):12929‐12940. 31490085 10.1096/fj.201900887RR PMC6902740

[41] Yi Z , Deng M , Scott MJ , et al. Immune‐responsive gene 1/itaconate activates nuclear factor erythroid 2‐related factor 2 in hepatocytes to protect against liver ischemia‐reperfusion injury. Hepatology. 2020;72(4):1394‐1411. 31997373 10.1002/hep.31147 PMC7702080

[42] Wang Z , Ka SO , Lee Y , Park BH , Bae EJ . Butein induction of HO‐1 by p38 MAPK/Nrf2 pathway in adipocytes attenuates high‐fat diet induced adipose hypertrophy in mice. Eur J Pharmacol. 2017;799:201‐210. 28213287 10.1016/j.ejphar.2017.02.021

[43] Taki‐Nakano N , Ohzeki H , Kotera J , Ohta H . Cytoprotective effects of 12‐oxo phytodienoic acid, a plant‐derived oxylipin jasmonate, on oxidative stress‐induced toxicity in human neuroblastoma SH‐SY5Y cells. Biochim Biophys Acta. 2014;1840(12):3413‐3422. 25219458 10.1016/j.bbagen.2014.09.003

[44] Zhao J , Liu L , Li X , et al. Neuroprotective effects of an Nrf2 agonist on high glucose‐induced damage in HT22 cells. Biol Res. 2019;52(1):53. 31542051 10.1186/s40659-019-0258-z PMC6754858

[45] Hunter DJ , March L , Chew M . Osteoarthritis in 2020 and beyond: a Lancet commission. Lancet. 2020;396(10264):1711‐1712. 33159851 10.1016/S0140-6736(20)32230-3

[46] Madzuki IN , Lau SF , Che Ahmad Tantowi NA , Mohd Ishak NI , Mohamed S . Labisia pumila prevented osteoarthritis cartilage degeneration by attenuating joint inflammation and collagen breakdown in postmenopausal rat model. Inflammopharmacology. 2018;26(5):1207‐1217. 29460078 10.1007/s10787-018-0452-6

[47] Goldring MB , Otero M . Inflammation in osteoarthritis. Curr Opin Rheumatol. 2011;23(5):471‐478. 21788902 10.1097/BOR.0b013e328349c2b1 PMC3937875

[48] Beyer K , Partecke LI , Roetz F , et al. LPS promotes resistance to TRAIL‐induced apoptosis in pancreatic cancer. Infect Agent Cancer. 2017;12:30. 28572836 10.1186/s13027-017-0139-4 PMC5450120

[49] Liu M , Zhang J , Liu W , Wang W . Salidroside protects ATDC5 cells against lipopolysaccharide‐induced injury through up‐regulation of microRNA‐145 in osteoarthritis. Int Immunopharmacol. 2019;67:441‐448. 30586667 10.1016/j.intimp.2018.12.041

[50] Lei J , Fu Y , Zhuang Y , Zhang K . Sema4D aggravated LPS‐induced injury via activation of the MAPK signaling pathway in ATDC5 chondrocytes. Biomed Res Int. 2020;2020:8691534. 32382577 10.1155/2020/8691534 PMC7196969

[51] Liu Q , Wu Z , Hu D , Zhang L , Wang L , Liu G . Low dose of indomethacin and hedgehog signaling inhibitor administration synergistically attenuates cartilage damage in osteoarthritis by controlling chondrocytes pyroptosis. Gene. 2019;712:143959. 31278964 10.1016/j.gene.2019.143959

[52] Sun K , Luo J , Jing X , et al. Hyperoside ameliorates the progression of osteoarthritis: an in vitro and in vivo study. Phytomedicine. 2021;80:153387. 33130473 10.1016/j.phymed.2020.153387

[53] Gao F , Zhang S . Loratadine alleviates advanced glycation end product‐induced activation of NLRP3 inflammasome in human chondrocytes. Drug Des Devel Ther. 2020;14:2899‐2908. 10.2147/DDDT.S243512 PMC7382759 32801633

[54] Houghton CA , Fassett RG , Coombes JS . Sulforaphane and other nutrigenomic Nrf2 activators: can the clinician's expectation be matched by the reality? Oxid Med Cell Longev. 2016;2016:7857186. 26881038 10.1155/2016/7857186 PMC4736808

[55] Crunkhorn S . Deal watch: ABBOTT boosts investment in NRF2 activators for reducing oxidative stress. Nat Rev Drug Discov. 2012;11(2):96. 10.1038/nrd3655 22293557

